# Acupuncture for Impaired Glucose Tolerance in People With Obesity: A Protocol for a Multicenter Randomized Controlled Trial

**DOI:** 10.3389/fmed.2022.932102

**Published:** 2022-07-12

**Authors:** Yan Yan, Yuanjie Sun, Xinlu Wang, Lili Zhu, Yu Chen, Zhishun Liu

**Affiliations:** ^1^Department of Acupuncture, Guang’anmen Hospital, China Academy of Chinese Medical Sciences, Beijing, China; ^2^New Zealand College of Chinese Medicine, Auckland, New Zealand

**Keywords:** overweight, obesity, impaired glucose tolerance, randomized controlled trial, acupuncture, protocol

## Abstract

**Background:**

Impaired glucose tolerance (IGT) is associated with being overweight/obesity and is a powerful risk factor for the disease of diabetes. In addition to lifestyle intervention that shows limited clinical application, acupuncture treatment has been a feasible treatment method for IGT in clinical practice. However, the effectiveness of acupuncture treatment has not been proved in evidence-based practice. Therefore, we design a multicenter randomized controlled trial to evaluate the efficacy and safety of acupuncture treatment for IGT in people with overweight/obesity.

**Methods:**

The trial will be conducted at hospitals in three different sites in China. A total of 196 participants will be recruited and randomly assigned at a ratio of 1:1 to either to the acupuncture group or the sham acupuncture (SA) group. Both groups will receive 30 sessions of treatment for 12 consecutive weeks and will be provided with lifestyle intervention and a 24-week follow-up. The primary outcome will be change in the baseline value of 2-h blood glucose (2hPG) on the 12th week. Additionally, the expectancy of acupuncture, blinding, and safety will also be assessed. All statistical analyses will be performed by two-sided test, and a *p*-value of less than 0.05 will be considered statically significant.

**Discussion:**

This study aims to provide quantitative clinical evidence of acupuncture effectiveness and safety in treating IGT in people who are overweight/obese.

**Clinical Trial Registration:**

[www.ClinicalTrials.gov], identifier [NCT05347030].

## Introduction

Impaired glucose tolerance (IGT), measured by oral glucose tolerance test (OGTT), is an intermediate stage of hyperglycemia between normoglycemia and diabetes (1, 2). The latest research shows that 541 million adults, or 10.6% of adults worldwide, are suffering from IGT (3), with China as the top epicenter. Owing to the similar pathophysiological mechanism of insulin resistance (4–6), IGT is more commonly seen in people who are overweight/obese. The prevalence of IGT in people who are overweight/obese in China reached 46.6% in 2008 (7). If no intervention is administered, every year, about 7% of individuals with IGT will develop diabetes (8), which is deeply associated with many chronic and acute diseases (3, 9, 10). Meanwhile, vascular complications associated with type 2 diabetes mellitus (T2DM) could occur in the IGT stage, indicating increased risks of cardiovascular and cerebrovascular diseases, microvascular diseases, tumors, and dementia in the future (11–15). Additionally, both pre-diabetes (including IGT) and obesity, like diabetes, have been determined as risk factors for critical coronavirus disease 2019 (COVID-19) (16–18). These diseases tend to cause a great damage to organs, warrant long-term interventions, and require large costs, posing a great challenge to public health across the globe (19).

Fortunately, the hyperglycemia in IGT is reversible (20), which means it is possible to prevent or delay the development of diabetes. At present, the recognized effective interventions include lifestyle modifications and pharmaceuticals.

Lifestyle modifications, including a healthy diet and more physical activity, are first-line treatments for patients with IGT who are overweight/obese (21–24). Research studies in different geographical areas globally have shown that lifestyle modifications reduce the risk of diabetes by more than 50% (8, 25, 26). However, it is difficult to apply the method concluded from lifestyle trials into clinical practice considering the high cost, considerable resources required, and lack of participant adherence to the intervention (27, 28). According to the Finnish Diabetes Prevention Study Group’s study, only 47% of the intervention group had a dietary fat intake of less than 30%, only 36% increased exercise intensity, and only 43% reached the weight loss target (26). Besides, patients with other diseases such as ischemic heart disease are excluded from this benefit, because their physical condition prevents them from achieving the recommended intensity of physical activity.

Several pharmaceuticals have been applied in treating IGT. Metformin was the first medicine shown to be effective (25), and it is also the only drug recommended by the American Diabetes Association for IGT treatment. However, Metformin is less effective than lifestyle modifications (31% vs. 58%), has adverse effects such as gastrointestinal reactions, and is not advised for all subgroups (25). The United States Food and Drug Administration has not approved it for preventive interventions (21). Acarbose is the only available medicine in China for treating IGT. Studies have shown that the risk of patients with IGT developing diabetes within 3.3 years is reduced by 25% (29), but many patients cannot tolerate its gastrointestinal side effects, and the drug is relatively expensive (30). As no sufficient evidence has shown that medication carries long-term efficacy and health economic benefits, relevant guidelines do not widely recommend medication intervention as the major measure to treat IGT.

Therefore, it is urgent to find a treatment method that not only treats IGT in people who are overweight/obese but is also characterized by the advantages of high safety, high compliance, good tolerance, and cost-efficiency.

As an important Traditional, Complementary, and Integrative Medicine (TCIM) therapy, acupuncture treatment has been more and more adopted in clinical practice all over the world. Its efficacy and safety have been preliminarily demonstrated in the treatment of insulin resistance (IR)-related diseases such as obesity, diabetes and its related complications, polycystic ovary syndrome, and hypertension (31–36). Although the physiological and pathological mechanisms of IGT are also related to IR (37, 38), the potential role of acupuncture in the treatment of obese people with IGT has not yet been determined by a well-designed randomized controlled trial (RCT). Therefore, this study is conducted to evaluate the efficacy and safety of acupuncture versus sham acupuncture (SA) on improving glucose metabolism among IGT people with Overweight/Obesity.

## Methods and Analysis

### Study Design

**FIGURE 1:**
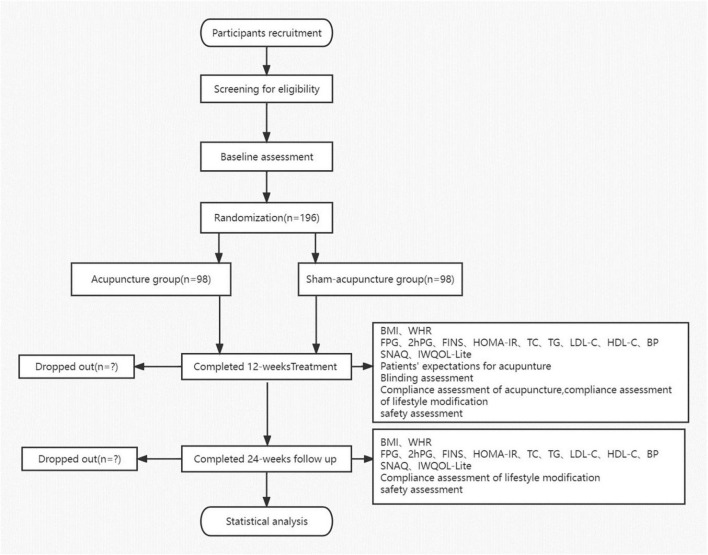
Flow chart. BMI, body mass index; WHR, waist-to-hip ratio; FPG, fasting plasma glucose; FINS, fasting serum insulin; HOMA-IR, homeostasis model assessment-insulin resistance index; TC, serum total cholesterol; TG, triglyceride; LDL-C, low-density lipoprotein cholesterol; HDL-C, high-density lipoprotein cholesterol; BP, blood pressure; IWQOL-Lite, Impact of Weight on Quality of Life-Lite Questionnaire; and SNAQ, Simplified Nutritional Appetite Questionnaire.

This is a prospective, multicenter, parallel-group, participant- and assessor-blinded randomized trial comprising a comparison between acupuncture and SA treatment for IGT in obese or overweight participants. All procedures and time frames are displayed in Figure 1 as per the guideline CONSORT.

### Participants

A total of 196 participants with IGT who are overweight/obese will be recruited from three different hospitals, Guang’anmen Hospital, Shenzhen Luohu District Traditional Chinese Medicine Hospital, and Yan Tai Hospital of Traditional Chinese Medicine, through public advertisements like posters, hospital websites, WeChat public accounts, and the Internet from October 2022 to December 2024. The research staff will introduce the background and process of the study in detail to potential subjects who contact the research team and express interest to participate. Once the informed consent form is signed, participants will be evaluated for eligibility by medical history inquiries, physical examinations, and necessary chemical examinations. The duration of the study for each participant will be 37 weeks, including 1-week baseline period, a 12-week treatment period, and a 24-week follow-up period.

### Diagnosis Criteria

The trial will adopt the diagnostic criteria of the World Health Organization [WHO (39)] for IGT (40) and the diagnostic criteria for overweight/obesity in the guidelines for the Prevention and Control of overweight and Obesity in Chinese Adults formulated by the Ministry of Health of China in 2003 (41, 42):

(1) IGT: fasting blood glucose (PG) is lower than 7 mmol/L, and 2-h blood glucose (2hPG) after oral 75 g glucose tolerance test (OGTT) is between 7.8 and ∼11.1 mmol/L.
(2) overweight: 24 < BMI ≤ 27.9, obesity: BMI ≥ 28, or BMI ≤ 24 but waist circumference ≥ 85 cm (men)/ ≥ 80 cm (women).

Note: The above two criteria should be met at the same time.

### Inclusion Criteria

Participants who meet all of the following criteria might be eligible: (1) those who meet the diagnostic criteria for overweight/obesity and IGT; (2) 18–60 years old; (3) those who can appropriately describe their wishes, voluntarily fill in the informed consent form, and agree to participate in clinical trials.

Note: The above three criteria should be met at the same time.

### Exclusion Criteria

Participants will be excluded if they meet any of the following criteria: (1) obesity secondary to heredity, drugs, and diseases; (2) those who have received weight-loss treatment or antidiabetic treatment including acupuncture, drugs, herbal medicine, and nutrient supplements in the past 3 months; (3) those who have taken drugs that have known effects on body weight or appetite in the past 3 months, such as corticosteroids, antidepressants, non-selective antihistamines *in vivo*, and nicotine substitutes; (4) IGT caused by abnormal thyroid function, endocrine tumors, or extensive liver damage; (5) abnormal glucose tolerance caused by thiazide diuretics, β-blockers, nicotinic acid drugs, quinolones, calcineurin inhibitors, interferon-alpha, etc.; (6) participants with serious primary diseases such as heart, lung, brain, liver, or hematopoietic system diseases, progressive malignant tumor, or other serious consumptive diseases; (7) participants with cognitive impairment and severe mental illness; (8) participants with blood coagulation dysfunction and who are scare of needles; (9) pregnant, breastfeeding, or planning to conceive within 37 weeks.

Note: Participants who meet any of the above criteria should be excluded.

### Randomization and Allocation Concealment

A random number will be generated with the SPSS software and concealed in opaque random envelopes. The envelopes will be prepared by a third party in advance, with the sequence number labeled on the surface and the random number and group allocation information sealed in. The random envelopes will be preserved by special personnel who are not involved in treatment and evaluation.

### Blinding

The participants will be blinded to the group allocation in this study, while the acupuncturists will not be blinded. The outcome will be evaluated by independent assessors who will be blinded to the allocation to minimize the bias associated with data collection. The statisticians will also be blinded to the allocation.

### Intervention

Study group: acupuncture + lifestyle recommendation; control group: sham acupuncture + lifestyle recommendation.

During the 12-week treatment, participants in both the acupuncture and SA groups will receive 30-session treatment, three times per week (once every other day) in the first six weeks and twice per week (with an interval of 2–3 days) in the last six weeks. Certified acupuncturists with no less than one year of clinical experience will perform the treatment. Acupuncture needles (Huatuo, Suzhou Medical Appliance) with the size of 0.3 mm × 40-75 mm will be used in the acupuncture group, while, acupuncture needles (Huatuo, Suzhou Medical Appliance) with the size of 0.2 mm × 25-40 mm will be used in the SA group. The participants in different groups will be treated separately to avoid contamination during the process.

### Acupuncture Group

**FIGURE 2:**
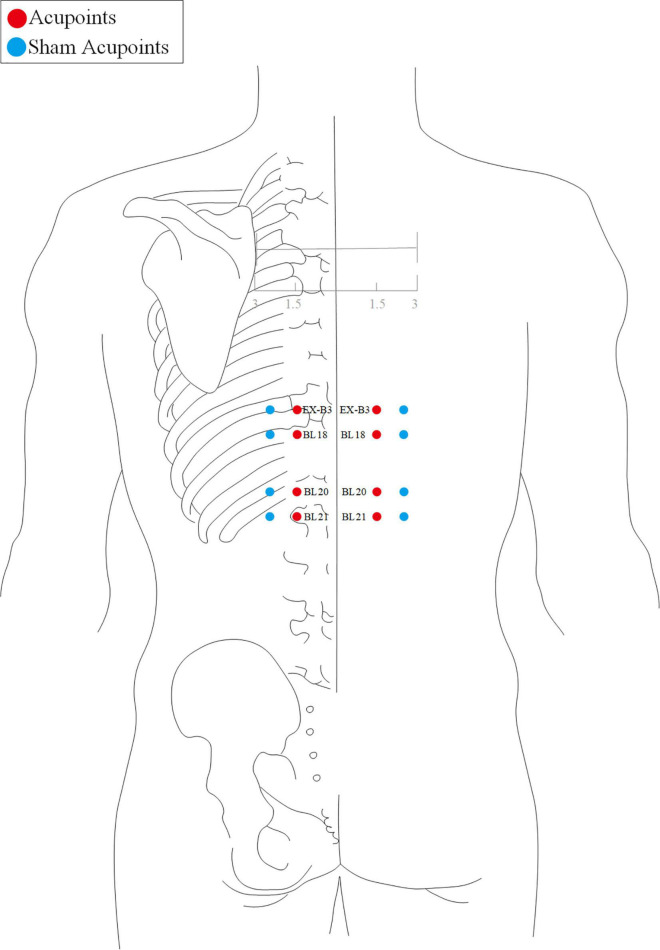
Stimulation points of the acupuncture group and the sham acupuncture group (back view).

**FIGURE 3:**
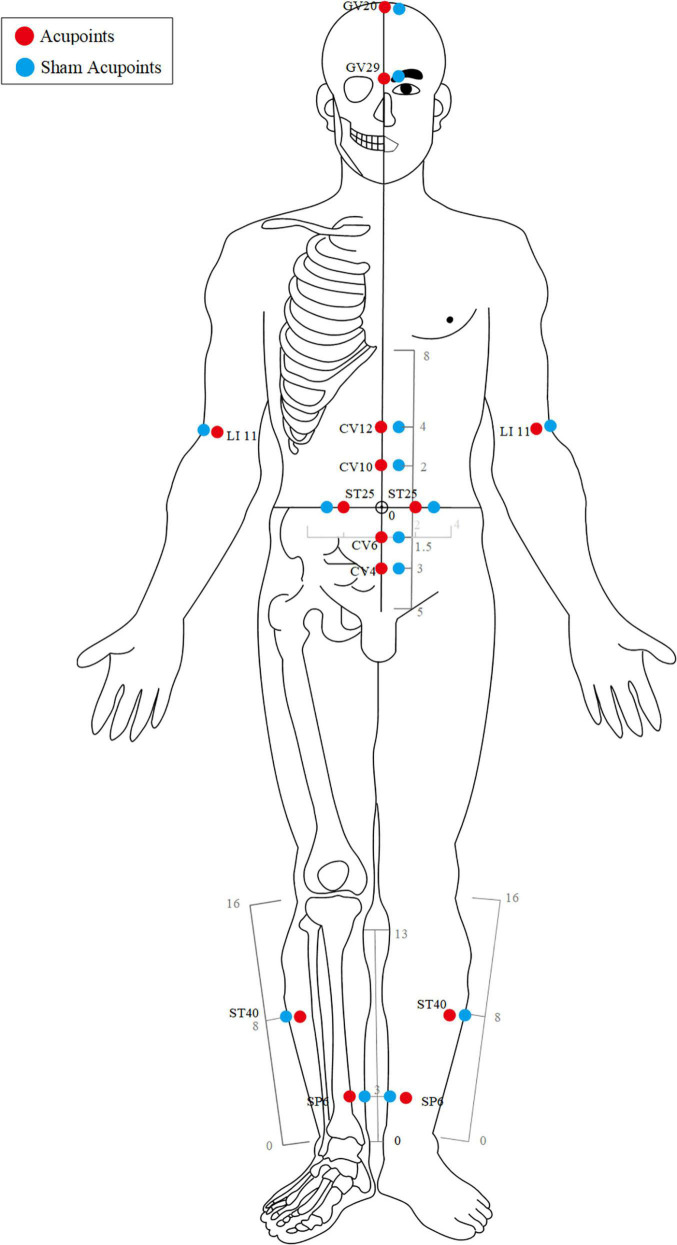
Stimulation points of the acupuncture group and the sham acupuncture group (front view).

The acupuncture point regimen is based on evidence-based clinical practice guidelines of traditional Chinese medicine (43, 44). All acupoints selected and their locations are displayed in Figures 2, 3. For acupoints EX-B3 (Yishu), BL18 (Gansu), BL20 (Pishu), and BL21 (Weishu), needles will be inserted at a depth of 15–25 mm depending on the participants’ somatotype and at an angle of 20° obliquely downward. The needles will be gently rotated and lifted three times to achieve a sense of sourness, distention, and heaviness (*de qi*), and will be withdrawn afterward immediately.

For GV20 (Baihui), the needle will be inserted backward under the cap membrane at an angle of 45° with the scalp; for GV29 (Yintang), the needle will be inserted downward at an angle of 15° under the periosteum; making the participant feel heavy pressure and acid distension. For acupoints LI11 (Quchi), CV12 (Zhongwan), CV10 (Xiawan), ST25 (Tianshu), CV6 (Qihai), CV4 (Guanyuan), ST40 (Fenglong), and SP6 (Sanyinjiao), needles will be inserted at a depth of 25–35 mm vertically. The needles will be gently rotated and lifted three times to achieve a sense of sourness, distention, and heaviness (de qi). The needles will be retained for 30 min with gentle lifting, thrusting, and twirling every 10 min during each session.

Acupoint location refers to the standard of acupoint location in the western Pacific region of the WHO and the name and location of acupoints (GB/T12346-2006).

### Sham Acupuncture Group

Participants in the SA group will receive minimal acupuncture at sham acupoints, which are located at a horizontal distance of one B-cun (≈ 20 mm) to the points used in the acupuncture group.

For sham EX-B3, BL18, BL20, and BL21, needles will be vertically inserted at a depth of 1–2 mm without any manipulation to avoid de-qi sensation and will be withdrawn immediately afterward.

For sham GV20, GV29, LI11, CV12, CV10, ST25, CV6, CV4, ST40, and SP6, needles will be vertically inserted at a depth of 1–2 mm without any manipulation to avoid the de-qi sensation. The needles will be retained for 30 min during each treatment session.

### Lifestyle Recommendation

Lifestyle recommendations will be made based on expert consensus and previous large-scale studies (45, 46). After randomization, participants in both the acupuncture and the SA groups will receive a brochure with lifestyle recommendations and a 20–30-min face-to-face communication with related specialists on the importance of a healthy lifestyle for the management of IGT and obesity. Specifically, the participants will be encouraged to lose 5∼10% of their initial weight *via* an integrated strategy of diet and exercise. They will be encouraged to limit calorie intake, reducing at least 400–500 kCal daily, to increase their activity gradually according to their individual situation, with the goal of over 30-min medium-or high-intensity physical activities such as brisk walking. Medium- or high-intensity physical activities are defined as reaching 50-70% of the maximum heart rate, being a little strenuous, and having fast breathing but no shortness of breath during exercise. The participants will be encouraged to avoid excessive alcohol intake and quit smoking. Whether these recommendations have been accepted and conducted will be evaluated during each visit.

### Rescue Medication

The participants will not be encouraged to receive any other treatment or medication for IGT or obesity/overweight during the study period to avoid any influence on the results. If participants receive any other treatment, they will also be included in this study, but all details will be recorded in the case report form.

If diabetes is diagnosed during the study period and regular hypoglycemic drugs are recommended by the specialists, the participants will be allowed to use hypoglycemic drugs for rescue. The details (name, dosage, frequency, start time, and duration of use) of drugs will be documented. The proportion of participants using rescue drugs in the groups will be compared.

### Outcome Measures

#### The Primary Outcome

The primary outcome will be change in the baseline value of 2hPG at the end of the 12-week treatment; 2hPG is the value of intravenous plasma glucose measured by 75 g OGTT, which is usually conducted to diagnose IGT and observe glucose metabolism (40). When the value of 2hPG fluctuates between 7.8 and 11.1 mmol/L, it will be regarded as IGT, and between 4.4 and 7.7 is the ideal control (40).

#### Secondary Outcomes

(1) Change from baseline value of 2-h blood glucose on weeks 24 and 36.

(2) Proportion of participants whose 2-h blood glucose dropped to normal from baseline on weeks 12, 24, and 36.

(3) Proportion of participants with at least 5% reductions (47) in body weight from baseline on weeks 4, 8, 12, 16, 24, and 36.

(4) Change from baseline of body mass index (BMI) and waist-to-hip ratio (WHR) on weeks 4, 8, 12, 16, 24, and 36.

(5) Change from baseline of fasting plasma glucose (FPG), fasting serum insulin (FINS), homeostasis model assessment-insulin resistance index (HOMA-IR), TC (serum total cholesterol), TG (triglyceride), LDL-C (low-density lipoprotein cholesterol), and high-density lipoprotein cholesterol (HDL-C) on weeks 12, 24, and 36.

(6) Change from baseline of blood pressure on weeks 12, 24, and 36.

(7) Change from baseline in the Impact of Weight on Quality of Life-Lite Questionnaire (IWQOL-Lite) scores on weeks 4, 8, 12, 16, 24, and 36.

IWQOL-Lite is an international scale used to measure an individual’s health-related quality of life (48). A total of 31 questions are divided into five subscales: 11 questions concerning physical function, 7 concerning self-esteem, 4 concerning sex life, 5 concerning public pressure, and 4 concerning work. The score of each question is divided into five grades, with 5 representing always, 4 often, 3 sometimes, 2 rarely, and 1 never. The total score of the questionnaire is 155, with higher score indicating worse quality of life. The MCID of IWQOL-Lite is 12 (49) for overweight/obesity.

(8) Change from baseline in the Simplified Nutritional Appetite Questionnaire (SNAQ) scores on weeks 4, 8, 12, 16, 24, and 36.

The SNAQ scale is a short and simple appetite assessment tool that can predict weight loss in adults (50). It measures the amount of food per meal, the number of meals per day, and the taste of the food. It consists of four items, each with five grades and with the total score ranging from 4 to 20 points. Lower scores indicate deterioration in appetite (50).

### Expectation Assessment

Participants’ expectations of acupuncture will be evaluated with reference to previous high-quality acupuncture clinical trials (51, 52). The participants will be assessed at baseline by the following two questions: “Do you think acupuncture will be effective in treating the illness?” and “Do you think acupuncture will be effective in controlling weight and decreasing glucose?” The response options will be “Ineffective,” “the effect is not good,” “uncertain,” “has some effect,” or “very effective.”

### Blinding Assessment

Blinding will be assessed using the James and Bang blinding indices (53). The participants will be told that they are 50% likely to be allocated to the traditional acupuncture group with deeper needling or to the modern acupuncture group with shallower needling. After the last treatment on week 12, each participant will be assessed with a questionnaire asking, “do you receive traditional acupuncture?,” with choice options of Yes or No.

### Compliance Assessment

(1) Compliance assessment of acupuncture

Compliance on acupuncture will be estimated by counting how many times the subjects have received the treatment, and the criterion for good compliance will be 80% and above. The calculation formula is as follows: compliance = (the number of times the subject has received treatment/the total number of times the subject should receive the treatment) × 100%.

(2) Compliance assessment of life recommendation

The participants lifestyle adjustment diary card, dietary change table, dietary compliance questionnaire, and exercise compliance scale were selected to evaluate the compliance on lifestyle recommendation.

The participant’s lifestyle adjustment diary card is a record of the completion of daily life guidance (complete “√,” unfinished “ × ,” to be checked every day). The dietary compliance questionnaire is extracted from the dietary treatment compliance questionnaire for patients with type 2 diabetes mellitus (54). It contains four questions concerning whether they eat strictly according to the dietary instruction plan, regular and quantitative meals, weigh or accurately measure food per meal, and use the Food Exchange scale to arrange their diet. Each question is provided with four options: it cannot be done at all, it can be done occasionally, it can be done basically, and it can be done completely.

The dietary change table before and after treatment included staple food and high-calorie non-staple food, and the options included “more than before treatment,” “more increase than before treatment,” “no change,” “less than before treatment,” and “more reduction than before treatment.”

The exercise compliance scale refers to the exercise compliance section of the coronary heart disease self-management scale (55). The question is “how did you participate in the recommended activities and exercise in the past month?” The options are: never participated in recommended activities (such as brisk walking, swimming, dancing, and Taijiquan), participated in recommended activities for less than 30 min per week, participated in recommended activities for 30–60 min per week, participated in recommended activities for 1–3 h a week, or participated in recommended activities for more than 3 h a week.

The specific outcome measures and time frame are displayed in Table 1.

**TABLE 1:** Study schedule.

Period	Run-in period	Treatment period			Follow-up period		
Day	7–0 days	4 weeks	8 weeks	12 weeks	16 weeks	24 weeks	36 weeks
Informed consent	√						
Inclusion/exclusion criteria	√						
Demography characteristics	√						
Medical history	√						
Anthropometry	√	√	√	√	√	√	√
Islet function	√			√		√	√
Blood lipid	√			√		√	√
Blood pressure	√			√		√	√
SNAQ	√	√	√	√	√	√	√
IWQOL-Lite	√	√	√	√	√	√	√
Expectation assessment	√						
Blindness assessment				√			
Combined use of drugs	√	√	√	√	√	√	√
Adverse events	√	√	√	√	√	√	√
Compliance assessment of acupuncture				√			
Compliance assessment of Lifestyle recommendation		√	√	√	√	√	√

*IWQOL-Lite, Impact of Weight on Quality of Life-Lite Questionnaire; SNAQ, Simplified Nutritional Appetite Questionnaire.*

### Safety Assessment

Adverse events (AEs) will be reported by the participants themselves and evaluated by outcome assessors and recorded in detail on AE forms throughout the study period. Based on their potential association with the intervention, AEs will be categorized by acupuncturists and related specialists as treatment-related or non-treatment-related within 24 h of occurrence. Treatment-related AEs include but not limited to broken needles, hematoma, infection, abscess around the location of the needle insertion, fainting, vomiting, nausea, palpitations, dizziness, anorexia, and insomnia that presented during or after acupuncture. Non-treatment-related AEs refer to any other unexpected AE that happened after the initiation of the study. According to the evaluation criteria of the NHS Committee (56), AEs will be divided into three levels (severe, moderate, and mild). Standardized procedures for AE management, especially for serious AEs, will be prepared, and all researchers and doctors involved will be trained.

### Quality Control

Before the trial, all staff will undergo special training on the purpose and content of the trial, treatment strategies, and quality requirements. Monitors will check the participant adverse event table, the participants’ lifestyle recommendation diary card, case report form, etc. once every week as well as the acupuncture operation during the treatment period. Dropouts and withdrawals and the underlying reasons will be documented in detail throughout the trial.

### Sample Size Calculation

In this study, change in 2hPG from baseline will be used as the basis for primary sample size estimation, based on the unpublished preliminary data collected from 10 participants in the acupuncture group and 10 SA group controls (-1.16 ± 0.42 mmol/L VS0.97 ± 0.34 mmol/L), and a sample size of 196 participants (98 per group) will need to provide the trial with 80% power with a two-sided significance level of 0.05 and 10% dropouts. The PASS 15 software was used for sample size calculation.

### Statistical Analysis

We will use the SPSS V.25 software (IBM Corp., Armonk, NY, United States) to perform all statistical analyses following the intention-to-treat principle. CI will be established at 95% and significance level at 0.05. Missing data will be calculated using the actual observational value without imputation if the dropout rate is no more than 5%. For continuous data, the data will be presented as mean ± *SD* if normally distributed or as median (IQR) if not normally distributed. Longitudinal continuous data will be compared between the groups by repeated-measures analysis of variance (ANOVA) including group and time–group interaction. Other continuous data will be analyzed by Student’s *t*-test and Wilcoxon rank-sum test, and categorical data by X ^2^ test or Fisher’s exact test, as appropriate. Linear correlation analysis will be conducted to measure the closeness of linear correlation between variables. Sensitivity analysis will be performed if necessary. A *p*-value < 0.05 will be considered statistically significant.

## Discussion

The disease of diabetes is a global killer and ranks among the top causes of premature death, with 1 in 10 adults living with it and almost half undiagnosed, and obesity is an independent risk factor. People with diabetes are more vulnerable to the COVID-19 and might develop more comorbidities if they are infected (3, 16–18). There is an apparent need to detect diabetes early and preferably to initiate an action during the IGT stage to reverse blood glucose to normal level through appropriate intervention. The principle of preventing the disease before onset is highly valued in the theories of traditional Chinese medicine (57). Stimulating specific points (acupoints) by acupuncture can induce the special sensation of “*de qi*” (such as soreness, numbness, heaviness, and distension) to regulate the circulation of *qi* through meridians, thus regulating viscera function and metabolism of glucose and lipid. Previous animal experiments have shown that the acupoints CV4 and SP6 can activate SIRT1/PGC-1α in skeletal muscle (37) and regulate the expression of key insulin signaling-related molecules, thus relieving insulin resistance in obese diabetic mice (58). Stimulating the acupoints CV12 and ST40 may influence SIRT1 in the hypothalamic arcuate nucleus, inducing an anorectic effect and reducing obesity with IR (59). Additionally, ST25 could also regulate the activity of glucose-inhibited neurons in the lateral hypothalamic area and improve the secretory function of adipose tissue to regulate glucose and lipid metabolism (60). This is the first randomized, multicenter and parallel trial to evaluate the efficacy and safety of acupuncture in improving glucose metabolism among IGT participants who are overweight or obese. It may provide valuable clinical evidence for the treatment of IGT and the prevention of diabetes.

However, some limitations of our study should be acknowledged. First, because of the characteristics of acupuncture, the acupuncturist will not be blinded in our trial, which may cause a potential bias. Second, the use of sham acupoints and minimal acupuncture without any manipulation may cause some biological effects leading to false-negative results. The settings of SA need considerations on how to avoid physiological effects as much as possible while blinding the participants. Acupuncture is a penetrating operation, and acupuncture marks will be left on participants’ body after the needle is withdrawn. Since most of the acupoints in this scheme are in the abdomen and limbs, it is convenient for participants to see whether any acupuncture marks are left after acupuncture. Therefore, to ensure blindness of the participants, we use sham acupoint and micro-acupuncture as a placebo control. However, it is difficult to avoid the non-placebo effect of sham needles, as a slight stimulus or a stronger stimulus of gentle touch may lead to different degrees of neurological responses.

## Ethics Statement

The studies involving human participants were reviewed and approved by Institutional Review Board of Guang’ammen Hospital. The participants provided their written informed consent to participate in this study.

## Author Contributions

ZL conceived the study, initiated the design, and revised the manuscript. YY participated in the design and drafted the manuscript. YS revised the important parts of this manuscript critically. XW and LZ participated in the revision of the manuscript. YC corrected the language problems in the manuscript. All authors contributed to the article and approved the submitted version.

## Conflict of Interest

The authors declare that the research was conducted in the absence of any commercial or financial relationships that could be construed as a potential conflict of interest.

## Publisher’s Note

All claims expressed in this article are solely those of the authors and do not necessarily represent those of their affiliated organizations, or those of the publisher, the editors and the reviewers. Any product that may be evaluated in this article, or claim that may be made by its manufacturer, is not guaranteed or endorsed by the publisher.

## References

[1] World Health Organization, IDF. Definition and Diagnosis of Diabetes Mellitus and Intermediate Hyperglycemia : Report of a WHO/IDF Consultation. (2006). Available online at: https://www.who.int/publications/i/item/definition-and-diagnosis-of-diabetes-mellitus-and-intermediate-hyperglycaemia (accessed June 21, 2022).

[2] American Diabetes Association. 2. Classification and diagnosis of diabetes: standards of medical care in diabetes-2021. *Diabetes Care.* (2021) 44 (Suppl. 1):S15–33. 10.2337/dc21-S002 33298413

[3] IDF Diabetes Atlas. *International Diabetes Federation.* (2022). Available online at: https://diabetesatlas.org/ (accessed June 21, 2022).

[4] Kahn SE Hull RL Utzschneider KM. Mechanisms linking obesity to insulin resistance and type 2 diabetes. *Nature.* (2006) 444:840–6. 10.1038/nature05482 17167471

[5] Attallah H Friedlander AL Hoffman AR. Visceral obesity, impaired glucose tolerance, metabolic syndrome, and growth hormone therapy. *Growth Horm IGF Res.* (2006) 16 (Suppl. A):S62–7. 10.1016/j.ghir.2006.03.004 16624603

[6] Reaven GM. The role of insulin resistance and hyperinsulinemia in coronary heart disease. *Metabolism.* (1992) 41 (5 Suppl. 1):16–9. 10.1016/0026-0495(92)90088-R 1574008

[7] Yang W Lu J Weng J Jia W Ji L Xiao J Prevalence of diabetes among men and women in China. *N Engl J Med.* (2010) 362:1090–101. 10.1056/NEJMoa0908292 20335585

[8] Li G Zhang P Wang J Gregg EW Yang W Gong Q The long-term effect of lifestyle interventions to prevent diabetes in the China Da Qing diabetes prevention study: a 20-year follow-up study. *Lancet.* (2008) 371:1783–9. 10.1016/s0140-6736(08)60766-7 18502303

[9] O’Leary K. Diabetes risk after COVID-19. *Nat Med.* (2022). 10.1038/d41591-022-00047-7 [Epub ahead of print]. 35347319

[10] Rubino F Amiel SA Zimmet P Alberti G Bornstein S Eckel RH New-onset diabetes in covid-19. *N Engl J Med.* (2020) 383:789–90. 10.1056/NEJMc2018688 32530585 PMC7304415

[11] Huang Y Cai X Mai W Li M Hu Y. Association between prediabetes and risk of cardiovascular disease and all cause mortality: systematic review and meta-analysis. *BMJ.* (2016) 355:i5953. 10.1136/bmj.i5953 PMC5121106 27881363

[12] Lamparter J Raum P Pfeiffer N Peto T Höhn R Elflein H Prevalence and associations of diabetic retinopathy in a large cohort of prediabetic subjects: the Gutenberg health study. *J Diabetes Complicat.* (2014) 28:482–7. 10.1016/j.jdiacomp.2014.02.008 24630763

[13] Zhou XH Qiao Q Zethelius B Pyörälä K Söderberg S Pajak A Diabetes, prediabetes and cancer mortality. *Diabetologia.* (2010) 53:1867–76. 10.1007/s00125-010-1796-7 20490448

[14] Ohara T Doi Y Ninomiya T Hirakawa Y Hata J Iwaki T Glucose tolerance status and risk of dementia in the community: the Hisayama study. *Neurology.* (2011) 77:1126–34. 10.1212/WNL.0b013e31822f0435 21931106

[15] Chen S Zhang Q Dai G Hu J Zhu C Su L Association of depression with pre-diabetes, undiagnosed diabetes, and previously diagnosed diabetes: a meta-analysis. *Endocrine.* (2016) 53:35–46. 10.1007/s12020-016-0869-x 26832340

[16] Vargas-Vázquez A Bello-Chavolla OY Ortiz-Brizuela E Campos-Muñoz A Mehta R Villanueva-Reza M Impact of undiagnosed type 2 diabetes and pre-diabetes on severity and mortality for SARS-CoV-2 infection. *BMJ Open Diabetes Res Care.* (2021) 9:e002026. 10.1136/bmjdrc-2020-002026 33593750 PMC7887863

[17] Sosibo AM Khathi A. Pre-diabetes and COVID-19, could we be missing the silent killer? *Exp Biol Med.* (2021) 246:369–70. 10.1177/1535370220973451 33215530 PMC7885044

[18] Kass DA Duggal P Cingolani O. Obesity could shift severe COVID-19 disease to younger ages. *Lancet.* (2020) 395:1544–5. 10.1016/s0140-6736(20)31024-2 32380044 PMC7196905

[19] Echouffo-Tcheugui JB Selvin E. Prediabetes and what it means: the epidemiological evidence. *Annu Rev Public Health.* (2021) 42:59–77. 10.1146/annurev-publhealth-090419-102644 33355476 PMC8026645

[20] Söderberg S Zimmet P Tuomilehto J de Courten M Dowse GK Chitson P High incidence of type 2 diabetes and increasing conversion rates from impaired fasting glucose and impaired glucose tolerance to diabetes in Mauritius. *J Intern Med.* (2004) 256:37–47. 10.1111/j.1365-2796.2004.01336.x 15189364

[21] US Preventive Services Task Force, Davidson KW Barry MJ Mangione CM Cabana M Caughey AB Screening for prediabetes and type 2 diabetes: US preventive services task force recommendation statement. *JAMA.* (2021) 326:736–43. 10.1001/jama.2021.12531 34427594

[22] Cosentino F Grant PJ Aboyans V Bailey CJ Ceriello A Delgado V 2019 ESC guidelines on diabetes, pre-diabetes, and cardiovascular diseases developed in collaboration with the EASD. *Eur Heart J.* (2020) 41:255–323. 10.1093/eurheartj/ehz486 31497854

[23] WHO. *Global Report on Diabetes.* (2016). Available online at: https://www.who.int/publications/i/item/9789241565257 (accessed June 21, 2022).

[24] Paulweber B Valensi P Lindström J Lalic NM Greaves CJ McKee M A European evidence-based guideline for the prevention of type 2 diabetes. *Horm Metab Res.* (2010) 42(Suppl. 1):S3–36. 10.1055/s-0029-1240928 20391306

[25] Knowler WC Barrett-Connor E Fowler SE Hamman RF Lachin JM Walker EA Reduction in the incidence of type 2 diabetes with lifestyle intervention or metformin. *N Engl J Med.* (2002) 346:393–403. 10.1056/NEJMoa012512 11832527 PMC1370926

[26] Tuomilehto J Lindström J Eriksson J Valle T Hämäläinen H Ilanne-Parikka P Prevention of type 2 diabetes mellitus by changes in lifestyle among subjects with impaired glucose tolerance. *N Engl J Med.* (2001) 344:1343–50. 10.1056/nejm200105033441801 11333990

[27] Tuomilehto J Schwarz P Lindström J. Long-term benefits from lifestyle interventions for type 2 diabetes prevention: time to expand the efforts. *Diabetes Care.* (2011) 34(Suppl. 2):S210–4. 10.2337/dc11-s222 21525457 PMC3632163

[28] Eddy DM Schlessinger L Kahn R. Clinical outcomes and cost-effectiveness of strategies for managing people at high risk for diabetes. *Ann Intern Med.* (2005) 143:251–64. 10.7326/0003-4819-143-4-200508160-00006 16103469

[29] Chiasson JL Josse RG Gomis R Hanefeld M Karasik A Laakso M. Acarbose for prevention of type 2 diabetes mellitus: the STOP-NIDDM randomised trial. *Lancet.* (2002) 359:2072–7. 10.1016/s0140-6736(02)08905-5 12086760

[30] Nathan DM Davidson MB DeFronzo RA Heine RJ Henry RR Pratley R Impaired fasting glucose and impaired glucose tolerance: implications for care. *Diabetes Care.* (2007) 30:753–9. 10.2337/dc07-9920 17327355

[31] Sui Y Zhao HL Wong VC Brown N Li XL Kwan AK A systematic review on use of Chinese medicine and acupuncture for treatment of obesity. *Obes Rev.* (2012) 13:409–30. 10.1111/j.1467-789X.2011.00979.x 22292480

[32] Li SQ Chen JR Liu ML Wang YP Zhou X Sun X. Effect and safety of acupuncture for type 2 diabetes mellitus: a systematic review and meta-analysis of 21 randomised controlled trials. *Chin J Integr Med.* (2021) 1–9. 10.1007/s11655-021-3450-2 34432205

[33] Meyer-Hamme G Friedemann T Greten J Gerloff C Schroeder S. Electrophysiologically verified effects of acupuncture on diabetic peripheral neuropathy in type 2 diabetes: the randomized, partially double-blinded, controlled ACUDIN trial. *J Diabetes.* (2021) 13:469–81. 10.1111/1753-0407.13130 33150711

[34] Zheng R Qing P Han M Song J Hu M Ma H The effect of acupuncture on glucose metabolism and lipid profiles in patients with PCOS: a systematic review and meta-analysis of randomized controlled trials. *Evid Based Complement Alternat Med.* (2021) 2021:5555028. 10.1155/2021/5555028 33824676 PMC8007365

[35] Yang J Chen J Yang M Yu S Ying L Liu GJ Acupuncture for hypertension. *Cochrane Database Syst Rev.* (2018) 11:Cd008821. 10.1002/14651858.CD008821.pub2 30480757 PMC6516840

[36] Jung H Yeo S Lim S. Effects of acupuncture on cardiovascular risks in patients with hypertension: a Korean cohort study. *Acupunct Med.* (2021) 39:116–25. 10.1177/0964528420920290 32567334

[37] Liang F Chen R Nakagawa A Nishizawa M Tsuda S Wang H Low-frequency electroacupuncture improves insulin sensitivity in obese diabetic mice through activation of SIRT1/PGC-1α in skeletal muscle. *Evid Based Complement Alternat Med.* (2011) 2011:735297. 10.1155/2011/735297 20981161 PMC2964507

[38] Abdul-Ghani MA Jenkinson CP Richardson DK Tripathy D DeFronzo RA. Insulin secretion and action in subjects with impaired fasting glucose and impaired glucose tolerance: results from the veterans administration genetic epidemiology study. *Diabetes.* (2006) 55:1430–5. 10.2337/db05-1200 16644701

[39] World Health Organization. *Definition, Diagnosis and Classification of Diabetes Mellitus and its Complications: Report of a WHO Consultation. Part 1, Diagnosis and Classification of Diabetes Mellitus. No. WHO/NCD/NCS/99.2.* (1999). Available online at: https://apps.who.int/iris/bitstream/handle/10665/66040/?sequence=1 (accessed June 21, 2022).

[40] Alberti K Zimmet PZ. Definition, diagnosis and classification of diabetes mellitus and its complications. Part 1: diagnosis and classification of diabetes mellitus provisional report of a WHO consultation. *Diabet Med.* (1998) 15:539–53. 10.1002/(SICI)1096-9136(199807)15:7<539::AID-DIA668>3.0.CO;2-S 9686693

[41] Department of Disease Control, Ministry of Health of the People’s Republic of China. *Guidelines for the Prevention and Control of Overweight and Obesity in Chinese Adults.* Beijing: People’s Medical Publishing House (2006).

[42] WHO Expert Consultation. Appropriate body-mass index for Asian populations and its implications for policy and intervention strategies. *Lancet.* (2004) 363:157–63. 10.1016/s0140-6736(03)15268-3 14726171

[43] China Association of Acupuncture-Moxibustion. *Evidence-Based Guidelines of Clinical Practice with Acupuncture and Moxibustion: Simple Obesity.* Beijing: China Press of Traditional Chinese Medicine (2015).

[44] Fang CH Tong XL Duan JG Ni Q Wei JP Xie CG Guide to evidence-based clinical practice of traditional Chinese medicine in pre-diabetes mellitus. *J Tradit Chin Med.* (2017) 58:5.

[45] Chinese Society of Endocrinology, Chinese Diabetes Society, Chinese Endocrinologist Association, Endocrine and Metabolic Disease Branch of Chinese Research Hospital Association, Diabetes Branch of Chinese Research Hospital Association. Intervention for adults with pre-diabetes: a Chinese expert consensus. *Chin J Endocrinol Metab.* (2020) 36:371–80. 10.3760/cma.j.cn311282-20200115-00022 30704229

[46] The Diabetes Prevention Program Research Group. The diabetes prevention program. Design and methods for a clinical trial in the prevention of type 2 diabetes. *Diabetes Care.* (1999) 22:623–34. 10.2337/diacare.22.4.623 10189543 PMC1351026

[47] Jensen MD Ryan DH Apovian CM Ard JD Comuzzie AG Donato KA 2013 AHA/ACC/TOS guideline for the management of overweight and obesity in adults: a report of the American college of cardiology/American heart association task force on practice guidelines and the obesity society. *Circulation.* (2014) 129 (25 Suppl. 2):S102–38. 10.1161/01.cir.0000437739.71477.ee 24222017 PMC5819889

[48] Kolotkin R Crosby R Kosloski K Williams G. Development of a brief measure to assess quality of life in obesity. *Obes Res.* (2001) 9:102–11. 10.1038/oby.2001.13 11316344

[49] Warkentin LM Majumdar SR Johnson JA Agborsangaya CB Rueda-Clausen CF Sharma AM Weight loss required by the severely obese to achieve clinically important differences in health-related quality of life: two-year prospective cohort study. *BMC Med.* (2014) 12:175. 10.1186/s12916-014-0175-5 25315502 PMC4212133

[50] Wilson MM Thomas DR Rubenstein LZ Chibnall JT Anderson S Baxi A Appetite assessment: simple appetite questionnaire predicts weight loss in community-dwelling adults and nursing home residents. *Am J Clin Nutr.* (2005) 82:1074–81. 10.1093/ajcn/82.5.1074 16280441

[51] Sun Y Liu Y Liu B Zhou K Yue Z Zhang W Efficacy of acupuncture for chronic prostatitis/chronic pelvic pain syndrome : a randomized trial. *Ann Intern Med.* (2021) 174:1357–66. 10.7326/m21-1814 34399062

[52] Sun Y Liu Y Chen H Yan Y Liu Z. Electroacupuncture for stress-predominant mixed urinary incontinence: a protocol for a three-armed randomised controlled trial. *BMJ Open.* (2021) 11:e038452. 10.1136/bmjopen-2020-038452 33414139 PMC7797267

[53] Bang H Ni L Davis CE. Assessment of blinding in clinical trials. *Control Clin Trials.* (2004) 25:143–56. 10.1016/j.cct.2003.10.016 15020033

[54] Li Y. *Study on the Influencing Factors of Dietary Treatment Compliance in Patients with Type 2 Diabetes Mellitus by Using the Theory of Planned Behavior.* Hebei: Hebei University (2017).

[55] Ren HY Tang P Zhao QH. Development and evaluation of coronary artery disease self-management scale. *J Third Mil Med Univ.* (2009) 11:4.

[56] HIS. *Learning From Adverse Events Through Reporting and Review: A National Framework for Scotland.* (2019). Available online at: https://www.healthcareimprovementscotland.org/our_work/governance_and_assurance/learning_from_adverse_events/national_framework.aspx (accessed June 21, 2022).

[57] Liang F Koya D. Acupuncture: is it effective for treatment of insulin resistance? *Diabetes Obes Metab.* (2010) 12:555–69. 10.1111/j.1463-1326.2009.01192.x 20590731

[58] Huang XY Zhang L Sun J Xu NG Yi W. Acupuncture alters expression of insulin signaling related molecules and improves insulin resistance in OLETF rats. *Evid Based Complement Alternat Med.* (2016) 2016:9651592. 10.1155/2016/9651592 27738449 PMC5055976

[59] Shu Q Chen L Wu S Li J Liu J Xiao L Acupuncture targeting SIRT1 in the hypothalamic arcuate nucleus can improve obesity in high-fat-diet-induced rats with insulin resistance *via* an anorectic effect. *Obes Facts.* (2020) 13:40–57. 10.1159/000503752 31935731 PMC7105640

[60] Yu Z Xia Y Ju C Shao Q Mao Z Gu Y Electroacupuncture regulates glucose-inhibited neurons in treatment of simple obesity. *Neural Regen Res.* (2013) 8:809–16. 10.3969/j.issn.1673-5374.2013.09.005 25206728 PMC4146081

